# Development of the automated immunomonitoring system

**DOI:** 10.1186/2051-1426-2-S3-P153

**Published:** 2014-11-06

**Authors:** Ryuji Osawa, Sachiko Yoshimura, Shoji Hisada, Koji Yoshida, Tsuyoshi Tojo, Hidenori Kuwata

**Affiliations:** 1 OncoTherapy Science, Inc., Kawasaki City, Japan; 2 Panasonic Healthcare Co., Ltd., Minato-ku, Japan

## 

Cancer vaccine which induces cytotoxic T lymphocyte targeting tumor associated antigens is one of major stream in cancer immunotherapy. Many cancer immunotherapy programs are in the clinical development all over the world and also the identification of biomarker for pharmacodynamics and patient selection should be considered.

Enzyme-linked immunospot (ELISPOT) assay is one useful method to detect antigen specific T cell response as an immunomonitoring. However, the standardization of the ELISPOT assay has not yet been established, and it has strongly been mandatory to detect accurate and objective immune response for clinical development of cancer vaccine. To overcome this problem, we have developed an automated immunomonitoring system, which performs cell culture processes including cell recovery, seeding and detection of the particular cells for ELISPOT assay. This automated cell culture system might be useful to control the quality of assays with cell culture and high-throughput outcomes. In this presentation, we show the concept and the characteristic of the automated immunomonitoring system.

